# Novel compound heterozygous *TMEM67* variants in a Vietnamese family with Joubert syndrome: a case report

**DOI:** 10.1186/s12881-020-0962-0

**Published:** 2020-01-30

**Authors:** Thi Phuong Hoa Bui, Ngoc Tu Nguyen, Van Doan Ngo, Hoai-Nghia Nguyen, Thi Thanh Ha Ly, Huy Duong Do, Minh-Tuan Huynh

**Affiliations:** 1 Medical Genetics Department, Vinmec Times City International Hospital-Times City, HaNoi, Vietnam; 2 Fetal Medicine Department, Vinmec Times City International Hospital-Times City, HaNoi, Vietnam; 3 Diagnostic Imaging Department, Vinmec Times City International Hospital-Times City, HaNoi, Vietnam; 4 Center for Molecular Medicine, University of Medicine and Pharmacy, Ho Chi Minh city, Vietnam

**Keywords:** Novel *TMEM67* splice-site variant, Joubert syndrome, Molar tooth sign, Whole exome sequencing

## Abstract

**Background:**

Joubert syndrome is a genetically heterogeneous autosomal recessive ciliopathy characterized by the combination of hypoplasia/aplasia of the cerebellar vermis, thickened and elongated superior cerebellar peduncles and a deep interpeduncular fossa, known as “molar tooth sign” associated with hypotonia, respiratory control disturbances and abnormal eye movements. To date, pathogenic variants in over 35 genes are known to cause autosomal recessive Joubert Syndrome, while one gene is associated with X-linked recessive inheritance.

**Case presentation:**

We describe here a non-consanguineous Vietnamese family with Joubert syndrome, a fetus and 10-year-old developmentally delayed boy. Ultrasonography showed ventriculomegaly at 26 + 6 weeks of gestation in the fetus. The 10-year-old-boy was diagnosed with cerebral palsy of unknown origin. Clinical physical examination at the age of 10, he showed clinical features of Joubert syndrome including typical facial dysmorphism, ataxia, severe psychomotor delay, oculomotor apraxia and molar tooth sign on brain MRI. Whole exome sequencing analysis identified a novel compound heterozygous c.725A > G p.Asn242Ser and c.313-3 T > G p.Lys105Valfs*16 *TMEM67* variant in the proband and the affected fetus. These two variants were inherited from each parent and confirmed by Sanger sequencing. The variant c.725A > G p.Asn242Ser was previously documented in patients with JS, the novel splice-site c.313-3 T > G p.Lys105Valfs*16 *TMEM67* variant produced an aberrant transcript with the loss of four nucleotides of exon 03.

**Conclusion:**

This study confirms the diagnosis of Joubert syndrome in a Vietnamese family and expands the mutational spectrum of *TMEM67* sequence variations. We also highlight the importance of molecular approaches to unravel underlying mechanisms of human genetic disorders. Early precise diagnosis could help provide further accurate genetic counseling for recurrence-risk assessment, future diagnostic option, management as well as treatment guidance for rare disorders.

## Background

Joubert syndrome (JS, MIM 213300) is an autosomal recessive ciliopathy characterized by specific midbrain-hindbrain malformations, recognizable on axial brain magnetic resonance imaging, known as the “Molar Tooth Sign”, hypotonia and developmental delays. Ciliopathies are a group of genetic disorders that are caused by abnormal formation or function of cellular primary cilia in many organs of the human body. To date, over 35 genes are known to cause autosomal recessive JS and one gene causes X-linked JS. Typical clinical features of JS include neonatal hypotonia, abnormal breathing patterns and eye movements, ataxia and developmental delays. Additionally, patients with JS also exhibit variable associated clinical features including retinal dystrophy, ocular colobomas, congenital heart disease, microcystic kidney disease, liver fibrosis, polydactyly, cleft clip and palate [1]. *TMEM67* (MIM *609884) encodes the Frizzled-like Wnt receptor, a transmembrane protein (meckelin) that regulates canonical Wnt/β-catenin signaling pathway in the developing cerebellum. *Tmem67*−/− mutant mice display cerebellar vermis hypoplasia/aplasia, deep interpeduncular fossa and posterior fossa defects compatible with JS phenotype [2]. Biallelic *TMEM67* sequence variations cause a wide range of clinical features observed in ciliopathies with multiorgan involvement and different clinical outcomes including JS (MIM 610688), Meckel-Gruber syndrome (MIM 607361), COACH syndrome (Cerebellar vermis hypoplasia, Oligophrenia, Ataxia, Coloboma and Hepatic fibrosis) (MIM 216360), RHYNS syndrome (Retinitis pigmentosa, Hypopituitarism, Nephronophthisis, Skeletal dysplasia), polycystic kidney disease, nephronophthisis-associated ciliopathy (MIM 613550) [3–7] (Table 1). Moreover, *TMEM67*-mutated patients with JS are also at increased risk for liver disease development complicated by probable portal hypertension in the second or third decades of life. The most relevant genotype-phenotype correlation has been established between *TMEM67* sequence variations and the subtypes of JS with liver disease [29]. We here describe the clinical characteristics and mutational analysis of the first Vietnamese family presenting clinical features of JS. Whole exome sequencing identified a novel compound heterozygous *TMEM67* variant. This study expands the mutational spectrum of *TMEM67* in JS as well as underscores the importance of molecular diagnosis and genetic counseling in patients initially diagnosed with cerebral palsy in Viet Nam.

**Table 1:** *TMEM67* sequence variations associated with a wide phenotype spectrum previously reported in the medical literature

*TMEM67* sequence variations (NM_153704)			Disease(s)	Reference(s)
c.DNA nomenclature	Protein change	Exon		
c.41G > A	p.Trp14*	E1	JS	[8]
c.175G > C	p.Ala59Pro	E2	CK and DPM*	[6]
c.245C > G	p.Pro82Arg	E2	JS	[4, 9]
c.270 T > G	p.Asn90Lys	E2	JS	[10]
c.274G > A	p.Gly92Arg	E2	MKS	[11]
c.297G > T	p.Lys99Asn	E2	JS, COACH	[4, 9]
c.300C > A	p.Cys100*	E2	JS, COACH	[4, 10]
c.329A > G	p.Asp110Gly	E3	JS	[12, 13]
c.370G > A	p.Glu124Lys	E3	JS	[10]
c.383_384delAC	p.His128fs*140	E3	MKS	[11]
c.387 T > A	p.Cys129*	E3	MKS	[10]
c.389C > G	p.Pro130Arg	E3	COACH	[4]
c.395G > C	p.Gly132Ala	E3	JS, COACH	[12, 14]
c.434 T > G	p.Leu145Trp	E4	COACH	[15]
c.442G > T	p.Ala184Ser	E4	JS	[12]
c.475 T > C	p.Ser159Pro	E4	JS	[12]
c.515G > A	p.Arg172Gln	E5	COACH	[4]
c.517 T > C	p.Cys173Arg	E5	JS	[16]
c.579delA	p.Gly195Aspfs*27	E6	MKS	[10]
c.579_580delAG	p.Gly195Ilefs*13	E6	JS	[9, 17]
c.622A > T	p.Arg208*	E6	RHYNS, MKS, JS, NPHP, ICHF, COACH	[4, 5, 7–9, 18–20]
c.641A > G	p.Tyr214Cys	E6	ICHF	[18]
c.647delA	p.Glu216fs*221	E6	MKS	[11]
c.675G > A	p.Trp225*	E8	COACH, MKS	[4, 10]
c.722C > G	p.Ala241Gly	E8	JS	[12]
c.725A > G	p.Asn242Ser	E8	JS, COACH	[4, 21, 22]
c.730A > G	p.Thr244Ala	E8	JS	[1]
c.748G > A	p.Gly250Arg	E8	JS	[9]
c.755 T > C	p.Met252Thr	E8	JS, MKS, NPHP	[4, 7, 9, 10, 20]
c.769A > G	p.Met257Val	E8	JS, COACH	[4, 9, 10]
c.797A > C	p.Asp266Ala	E8	JS	[8]
c.869G > T	p.Trp290Leu	E8	NPHP	[23]
c.903C > G	p.Asp301Glu	E9	JS	[10]
c.934 T > C	p.Ser312Pro	E9	JS	[16]
c.950C > G	p.Thr317Arg	E9	JS	[9, 17]
c.986A > C	p.Lys329Thr	E10	NPHP	[7]
c.1027 T > G	p.Phe343Val	E10	CK and DPM*	[6]
c.1045 T > C	p.Leu349Ser	E10	NPHP	[7]
c.1046 T > C	p.Leu349Ser	E10	COACH, MKS	[4, 10, 20]
c.1063C > T	p.Gln355*	E10	CK and DPM*	[6]
c.1073 T > C	p.Pro358Leu	E11	JS, COACH	[4, 10]
c.1077_1078del	p.Thr360Argfs*18	E11	JS	[10]
c.1081G > T	p.Glu361*	E11	JS, COACH	[4, 9]
c.1115C > A	p.Thr372Lys	E11	JS, COACH	[4, 8, 10]
c.1126C > G	p.Gln376Glu	E11	JS, COACH	[4, 9]
c.1127A > C	p.Gln376Pro	E11	MKS	[11]
c.1285C > T	p.Gln429*	E12	JS	[10]
c.1289A > G	p.Asp430Gly	E13	RHYNS, NPHP	[5, 19]
c.1319G > A	p.Arg440Gln	E13	MKS	[10, 20]
c.1321C > T	p.Arg441Cys	E13	COACH	[4]
c.1322G > T	p.Arg441Leu	E13	MKS	[10]
c.1336G > C	p.Asp446His	E13	MKS	[20]
c.1351C > T	p.Arg451*	E13	JS, NPHP, COACH	[4, 7, 9]
c.1387C > T	p.Arg463*	E13	NPHP	[7]
c.1392C > T	p.Arg441Cys	E13	MKS	[11]
c.1438A > G	p.Tyr513Cys	E15	COACH	[4]
c.1453C > T	p.Pro458Ser	E15	COACH	[4]
c.1536_1537del	p.Tyr513*	E15	JS	[12]
c.1538A > G	p.Tyr513Cys	E15	JS, COACH	[4, 9, 10, 24]
c.1538_1539delAT	p.Tyr513*	E15	MKS	[10]
c.1634G > A	p.Gly545Glu	E16	JS	[24]
c.1645C > T	p.Arg549Cys	E16	MKS	[11, 25]
c.1706G > A	p.Gly569Asp	E17	JS	[10]
c.1715C > T	p.Ala572Val	E17	CK and DPM*	[6]
c.1769 T > C	p.Phe590Ser	E17	JS	[10]
c.1675-?_2241 +?del	p.T559_Q747del	E17_E21	MKS	[20]
c.1843 T > C	p.Cys615Arg	E18	JS, COACH, NPHP, MKS	[4, 7, 9, 23, 26]
c.1847C > T	p.Ala616Val	E18	JS	[10]
c.1975 T > C	p.Ser659Pro	E20	JS, COACH	[4]
c.2002 T > C	p.Trp668Arg	E20	MKS	[10]
c.2018 T > C	p.Val673Ala	E20	NPHP	[7]
c.2086C > T	p.Leu696Phe	E20	JS	[1]
c.2290C > T	p.Arg764*	E22	JS	[12]
c.2301delT	p.Asp768Ilefs*5	E23	MKS	[10]
c.2311 T > C	p.Ser771Pro	E23	JS	[12]
c.2345A > G	p.His782Arg	E23	JS	[27]
c.2357G > A	p.Gly786Glu	E23	MKS	[10]
c.2368C > A	p.His790Asn	E23	JS	[1]
c.2413C > T	p.Arg805*	E23	JS, COACH	[4]
c.2439G > A	p.Ala813Ala	E23	MKS	[20]
c.2461G > A	p.Gly821Ser	E24	NPHP	[23]
c.2497 T > C	p.Ile833Thr	E24	COACH	[4]
c. 2498 T > C	p.Ile833Thr	E24	JS, COACH, NPHP	[4, 7–10, 19]
c.2522A > C	p.Gln841Pro	E24	JS, COACH	[4, 9, 12, 28]
c.2528A > G	p.Tyr843Cys	E24	MKS	[10]
c.2542G > T	p.Glu848*	E24	MKS	[10]
c.2557A > T	p.Lys853*	E25	MKS	[20]
c.2561dupA	p.Asn854Lysfs*5	E25	MKS	[10]
c.2689_2690insTA	p.Leu897Ilefs*64	E26	MKS	[10]
c.2758delT	p.Tyr920Thrfs*40	E26	JS, COACH	[14, 21, 22]
c.2801G > A	p.Gly934Glu	E27	JS	[1]
c.2802delA	p.Gly934Glyfs*26	E27	JS, COACH	[4, 9]
c.2825 T > G	p.Phe942Cys	E27	COACH	[4]
c.2879C > T	p.Ala960Val	E27	JS	[9]
c.2891C > T	p.Thr964Ile	E27	NPHP	[7]
c.3145C > T	p.Arg1049*	E28	COACH	[4]
c.3347C > T	p.Thr1116Met	E28	COACH	[4]

*Abbreviation*: *JS* Joubert syndrome, *MKS* Meckel-Gruber syndrome, *COACH* cerebellar vermis hypoplasia, Oligophrenia, Ataxia, Coloboma, Hepatic fibrosis, *RHYNS* Retinitis Pigmentosa, Hypopituitarism, Nephronophthisis, Skeletal dysplasia, *CK and DPM* Cystic kidneys and ductal plate malformations (*distinct prenatal form of nephronophthisis), *NPHP* Nephronophthisis, *ICHF* Isolated congenital hepatic fibrosis

## Case presentation

**Fig. 1:**
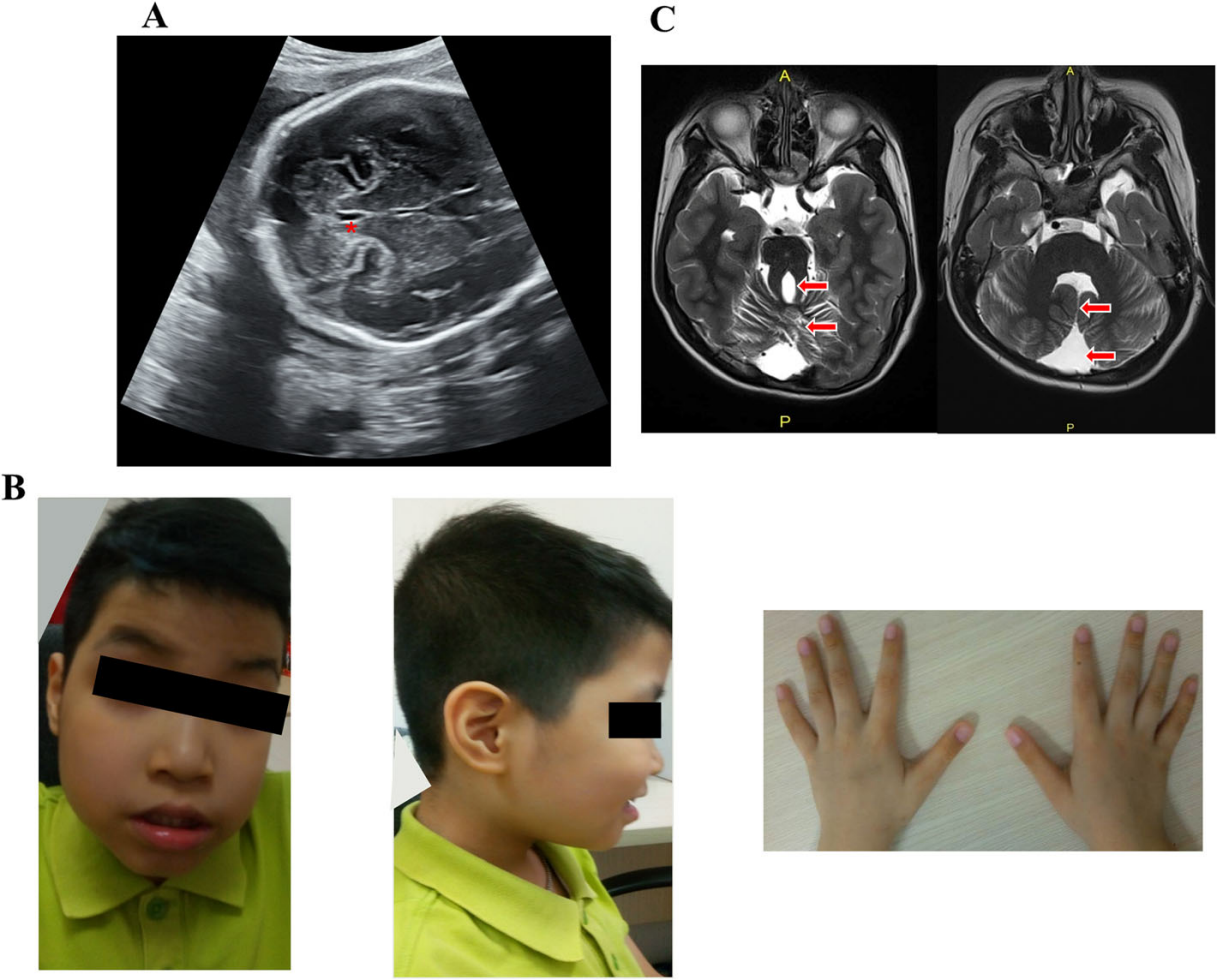
**a** Ultrasound at 26 + 6 weeks of gestation showing an abnormal enlarged fourth ventricle (red asterisk) and the ventricle floor is abnormal. **b** Photographs of the face (left image) and right profile (middle image) showing typical facial features of Joubert syndrome including prominent forehead, high arched eyebrows, bilateral ptosis, hypertelorism, lower lip eversion (left and middle image), mild clinodactyly of the fifth finger and tapered fingers (right image). **c** Axial MRI through the cerebellum and brain stem showing cerebellar vermis hypoplasia, thick and elongated superior cerebellar peduncles (red arrows, left image) and absence of the cerebellar vermis, deep interpeduncular fossa and the fourth ventricle has a bat-wing configuration (red arrows, right image)

This 38 year-old female first came to our clinic because of family history of developmental delay. The fetus (II:2) was the second child of a non-consanguineous Vietnamese healthy couple, mid-trimester prenatal ultrasound at 26 + 6 weeks of gestation showed an abnormal enlarged fourth ventricle with abnormalities of the ventricle floor (Fig. 1a). Moreover, renal hypoplasia and polycystic kidney were also noticed. She already had a first child (II:1) with developmental delay. This 10-year-old-boy was born at term after an uneventful pregnancy. His birth weight, height and head circumference were respectively 3400 g (50-90th centile), 53 cm (90th percentile), 34 cm (50th centile). Hypotonia and abnormal breathing pattern were noted at birth. He was diagnosed with cerebral palsy of unknown origin at 2 years old. Neither genetic counseling nor molecular genetic testing was provided. According to the clinical evaluation conducted at the age of 10, his weight, height and head circumference were respectively 23 kg (5th), 129 cm (5-10th) and 52 cm (10th–25th). The proband had severe psychomotor and language delay, began to walk at the age of 72 months and spoke the first word at the age of 60 months. Clinical physical evaluation showed a prominent forehead with bitemporal narrowing, high arched eyebrows, bilateral ptosis, hypertelorism, lower lip eversion, mild clinodactyly of the fifth finger and tapered fingers (Fig. 1b). The patient also displayed ataxic gait and oculomotor apraxia. He had mild intellectual disability. Family history was unremarkable. His MRI showed a pathognomonic finding of molar tooth sign (Fig. 1c). The couple was concerned about their pregnancy, and thus requested genetic counseling regarding her second pregnancy.

## Genetic analysis

### Conventional cytogenetics and array-CGH

Blood lymphocytes were cultured in RPMI 1640 supplemental with PHA (Gibco, USA) and chromosomes metaphases were harvested according to the laboratory standard protocol. Conventional cytogenetics showed a normal male karyotype 46,XY. 180 K array-CGH (Agilent Technologies, Santa Clara, USA) with a median probe spacing of 13 kb was carried out according to the manufacturer’s instructions and required at least three consecutive probes to make a call. Array-CGH analysis identified no chromosomal imbalances arr(1–22)×2,(X,Y)×1.

### Whole exome sequencing

**Fig. 2:**
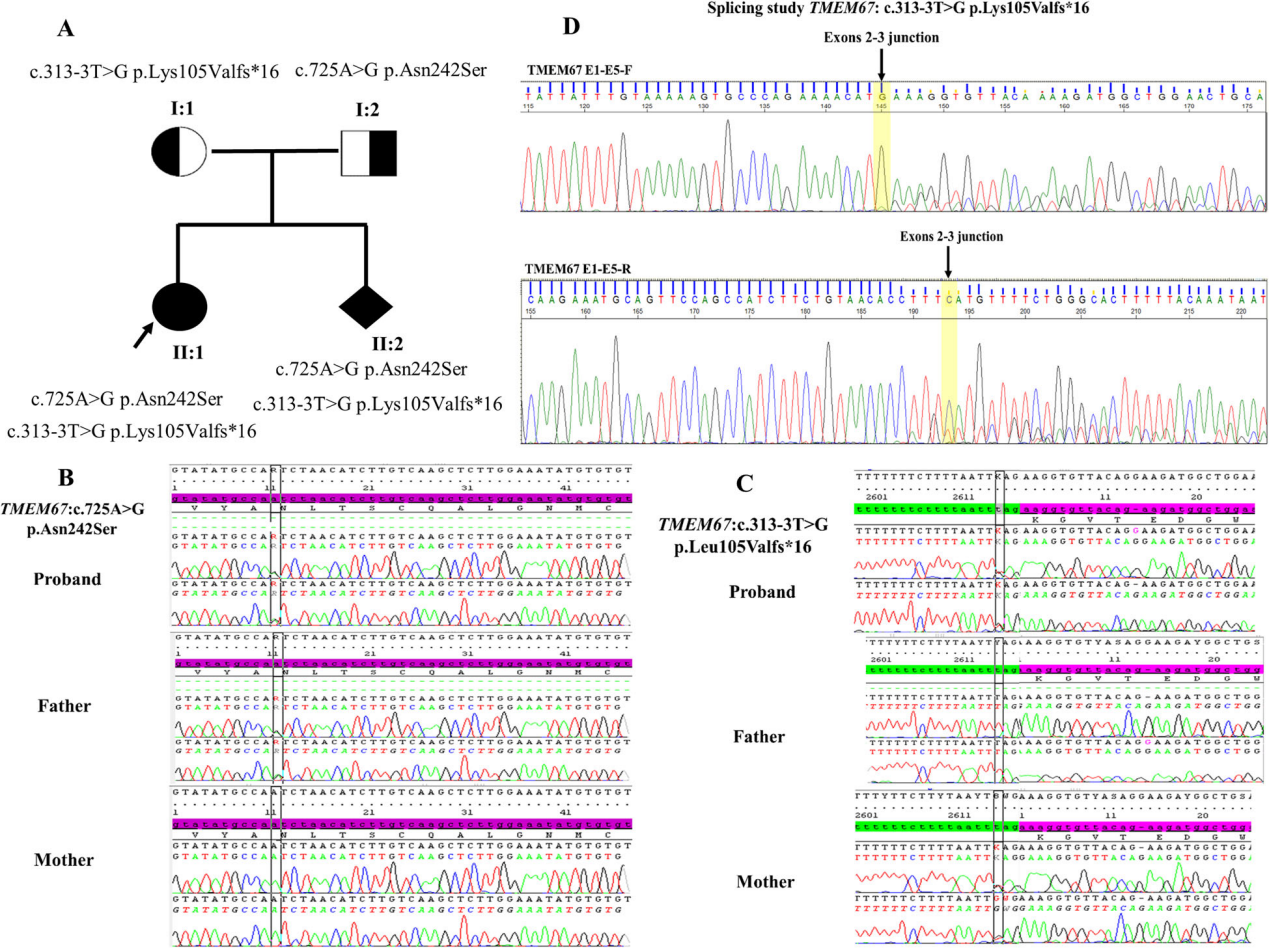
**a** Familial pedigree with Joubert syndrome shows a novel compound heterozygous *TMEM67* variant in the case-index and the fetus while the parents were heterozygous. **b**, **c** Sang sequencing showing the proband and his father were heterozygous for *TMEM67* c.725A > G p.Asn242Ser variant. Moreover, the proband and his mother were heterozygous for a novel *TMEM67* splice-site variant c.313-3 T > G p.Leu105Valfs*16. **d** Reverse transcriptase PCR showing alternative splicing effect with the deletion of 4 bps in exon 03 in the mRNA resulting in an aberrant transcript with premature codon stop. The last nucleotide in exon 2 is yellow highlighted

In order to find causative gene variants, we performed whole exome sequencing in the proband (II:1). Genomic DNA was extracted from whole blood from the family using standard methods (QIAGEN, Germany). Five hundred ng DNA was fragmented with a Biorupter (Diagenode, Seraing, Belgium) and the fragmented DNA quality was checked on a bioanalyser MultiNA (Shimadzu Corporation, Kyoto, Japan). Libraries were performed using the Ultra DNA library preparation kit (NewEngland, Biolabs, UK), exome enrichment was performed using TruSeq Exome Library Prep Kit (Illumina, USA) and IDT’s xGen® Exome Research Panel (Integrated DNA Technologies, USA). Finally, sequencing was done on an Illumina HiSeq® 2500. The exome was covered to a mean depth of 100, data with >10X mean coverage accounted for 95% of the whole data. Variants were validated by Sanger DNA sequencing using the ABI BigDye Terminator v3.1 Cycle Sequencing kit and the ABI PRISM 3130xl genetic analyzer (Applied Biosystems, CA, USA). Sequence alterations were reported according to Human Genome Variation Society guidelines (HGVS) and mapped to Human Genome Build GRCh37/UCSC hg19. Whole exome sequencing showed novel compound heterozygous c.725A > G p.Asn242Ser and c.313-3 T > G p.Lys105Valfs*16 *TMEM67* variants in the proband (II:1) (Fig. 2a). No other potentially pathogenic variants in other genes associated with developmental delay were identified. These two variants were interpreted as likely pathogenic according to standards and guidelines from the American College of Medical Genetics and Genomics. The variant c.725A > G has a MAF < 0.01 (PM2: Extremely low frequency). It was previously reported in patients with JS (PM3: For recessive disorders, detected in *trans* with a pathogenic variant and PP1:Co-segregation with disease in multiple affected family members) and predicted to be deleterious by in silico prediction (PP3: Multiple lines of computational evidence support a deleterious effect on the gene or gene product). The novel splice-site variant has never been documented (PM2: Absent from controls) and produces an aberrant splicing transcript (PVS1: Null variant). Sanger sequencing confirmed that the variant c.725A>G p.Asn242Ser was paternally inherited and the splice-site variant c.313-3T>G p.Lys105Valfs*16 was of maternal origin (Fig. 2b, c). Prenatal diagnosis was performed on genomic DNA extracted from amniocytes and Sanger sequencing identified the same compound heterozygous variant c.725A>G p.Asn242Ser and c.313-3T>G p.Lys105Valfs*16 in the fetus (II:2).

### Reverse transcriptase PCR for alternative splicing study (RT-PCR)

Total RNA from blood sample was extracted using Qiagen RNeasy blood mini kit according to the manufacturer’s instruction (Qiagen, Germany). One microgram of RNA was used for Reverse-Transcriptase PCR (RT-PCR) (Quantabio, USA). RT-PCR was performed with primers spanning exons 1 and 5. PCR products were separated on agarose gel 2% and sequenced using the ABI BigDye Terminator v.3.1 Cycle Sequencing kit and the ABIPRISM 3500 XL genetic analyzer (Applied Biosystems, CA, USA). Splicing study demonstrated that the novel splice-site variant produced an aberrant transcript with the loss of four nucleotides of exon 03 (Fig. 2d).

## Discussion and conclusion

In the era of clinical genomics in Viet Nam, clinical genetics testing is still relatively new and clinicians have very little knowledge about current approaches to genetic disorders. Many developmentally delayed children suffering from genetic disorders were diagnosed with cerebral palsy and they have not benefited from a modern multi-disciplinary care model. We report a Vietnamese family including a 10-year-old child diagnosed with cerebral palsy without etiologic diagnosis and a fetus with central nervous system malformations. Physical examination of the age of the 10-year old showed typical clinical features of JS and an MRI showing the pathognomonic finding of a molar tooth sign, which confirms the JS diagnosis. Whole exome sequencing identified compound heterozygous *TMEM67* variants in the proband (II:1). The variant c.725A > G p.Asn242Ser was previously documented in patients with JS and predicted to be pathogenic by SIFT, Polyphen-2 and Mutation Taster [21]. This variant was considered as founder mutation in Eastern Iranian population. The novel splice-site variant c.313-3 T > G p.Lys105Valfs*16 has never been documented in the medical literature and the nucleotide T at this position is highly conserved across multiple species (Additional file 1: Figure S1). Moreover, alternative splicing study demonstrated that the variant c.313-3 T > G p.Lys105Valfs*16 produced an aberrant transcript with the loss of the first four nucleotides of exon 03 leading to a premature stop codon.

*TMEM67* sequence variations were associated with a large clinical spectrum and sequence variants were distributed throughout the entire coding region (Table 1). However, several peculiar phenotypes might be predicted, i.e., *TMEM67* missense variants falling in exon 8 to 15, especially combined with a truncating variant would predict to give rise to Meckel-Gruber syndrome. In addition, most of *TMEM67* sequence variants were predominantly located in 8 of 28 exons (2, 6, 8, 11, 13, 15, 18, 24) [10]. Based on the review of *TMEM67* sequence variations previously recorded in the medical literature, our report also showed several mutational hotspots, which were consistent with the result documented by Lannicelli et al., 2010. The most *TMEM67* frequently mutated hotspot was exon 8, followed by exons 24, 18, 6, 13, 11, 2, 15 (Table 1).

Prenatal molecular diagnosis was carried out on DNA extracted from amniocytes and the fetus (II:2) harbored the same compound heterozygous *TMEM67* variants found in the 10-year-old sibling. The parents were referred for genetic counseling for the current pregnancy and the pregnancy was terminated at 32 weeks of gestation. Furthermore, the proband was recommended to follow annual surveillance of hepatic functions as well as evaluation of kidney function [30]. The couple was offered pre-implantation genetic diagnosis or prenatal diagnosis options for the future pregnancy. In conclusion, our study reports the first Vietnamese family of JS and expands the *TMEM67* mutational spectrum in JS. Furthermore, we also stress the important role of molecular approaches in order to identify the causative gene. Accurate diagnosis would further help in genetic counseling, early management of genetic disorders as well as offer prenatal diagnostic options for future pregnancy.

## Supplementary information

**Additional file 1:** **Figure S1.** Multiple sequence alignment of *TMEM67* sequences across species showing that the nucleotide c.313-3 T variant is well conserved throughout evolution (red box).

## Data Availability

The data was submitted in LOVD (Leiden Open Variation Database): https://databases.lovd.nl/shared/login. Individual number #00265632 in LOVD database.

## Abbreviations

Array-CGH: Array-comparative genomic hybridization
JS: Joubert syndrome
MRI: Magnetic resonance imaging
RT-PCR: Reverse-transcriptase polymerase chain reaction
SIFT: Sorting intolerant from tolerant
TMEM67: Transmembrane protein 67
