# Supplementary figures and images for: Novel compound heterozygous TMEM67 variants in a Vietnamese family with Joubert syndrome: a case report
Source: BMC Med Genet. 2020 Jan 30;21:18. doi: 10.1186/s12881-020-0962-0 (PMC6993522; doi:10.1186/s12881-020-0962-0)

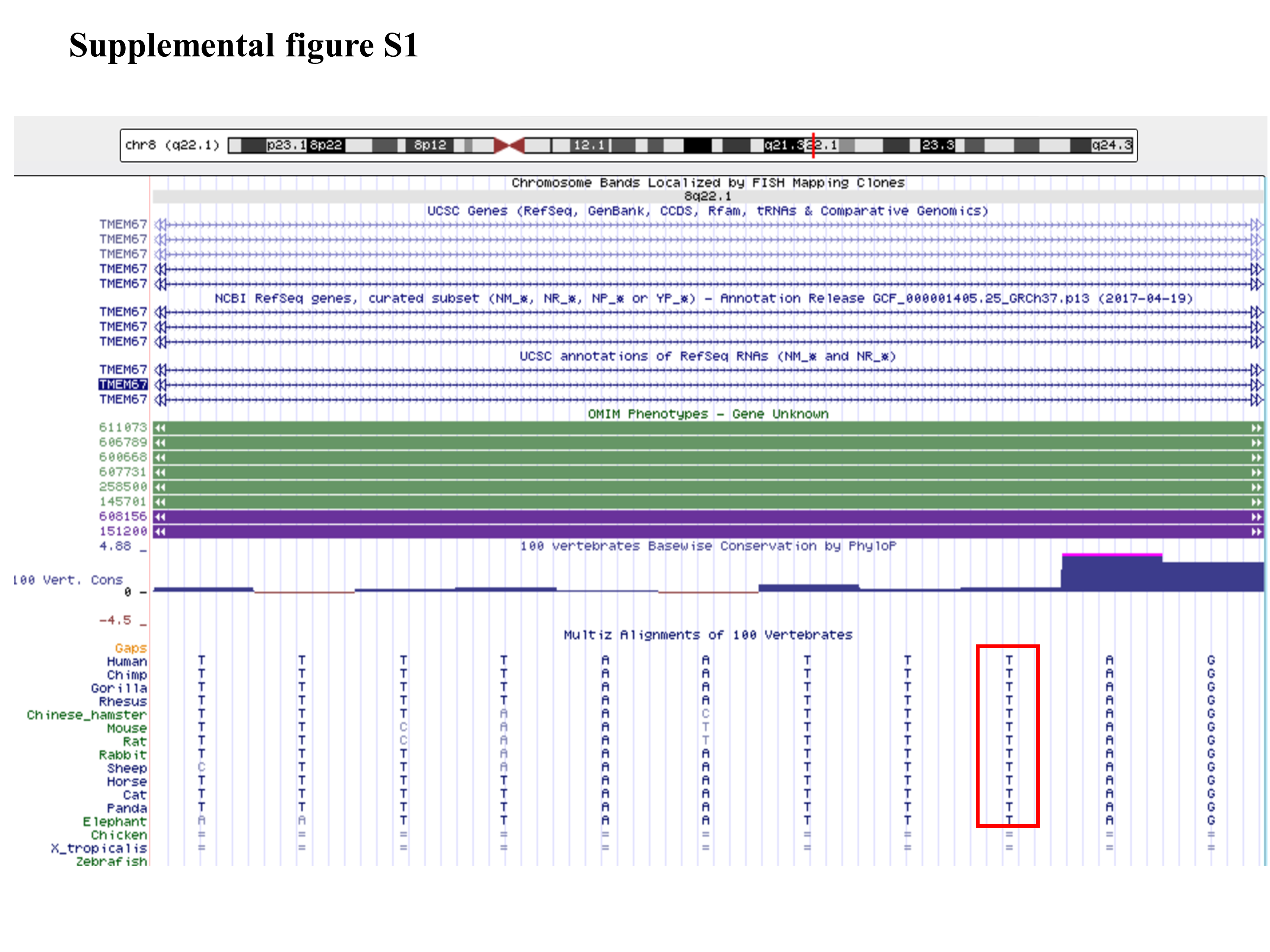

Supplement: Supplementary file 1 — Additional file 1: Figure S1. Multiple sequence alignment of TMEM67 sequences across species showing that the nucleotide c.313-3 T variant is well conserved throughout evolution (red box). [file 12881_2020_962_MOESM1_ESM.tif]
